# MicroRNA: unveiling novel mechanistic and theranostic pathways in diabetic cardiomyopathy

**DOI:** 10.3389/fphar.2025.1613844

**Published:** 2025-07-23

**Authors:** Akash De, Arnab Sarkar, Tanmoy Banerjee, Rudranil Bhowmik, Shuvam Sar, Md. Adil Shaharyar, Sanmoy Karmakar, Nilanjan Ghosh

**Affiliations:** ^1^ Bioequivalence Study Centre, Department of Pharmaceutical Technology, Jadavpur University, Kolkata, West Bengal, India; ^2^Molecular Pharmacology Research Laboratory, Department of Pharmaceutical Technology, Jadavpur University, Kolkata, West Bengal, India

**Keywords:** diabetic cardiomyopathy, miRNAs, theranostic approaches, mRNA therapy, clinical trials

## Abstract

Diabetic cardiomyopathy (DCM) is a prominent contributor to morbidity and mortality in people with diabetes worldwide. In diabetic patients, it is a chronic condition that is characterized by ventricular hypertrophy (VH), diastolic dysfunction, alteration of systolic function, and reduced ejection fraction, ultimately leading to heart failure (HF). Despite being extensively understood, the underlying causes of DCM remain obscure. Growing evidence has identified the contribution of microRNAs (miRNAs), a small non-coding RNA molecule playing a crucial part in the pathogenesis of DCM. These miRNAs have been linked with several mechanistic pathways involved in DCM, including inflammation, insulin resistance and cardiomyocyte apoptosis. miRNAs related to DCM include miR-9, 30d, 34a, 142-3p, 144, 150, 208a, etc. Thus, miRNAs present themselves as novel targets for diagnostic biomarkers and mechanistic therapeutics, which may prove to be clinically more efficient than other therapeutic approaches. This review highlights the role of miRNAs, which can act as the nodes of signalling networks that regulate the progression of DCM and also tries to decipher the complicated cross-talk between miRNAs and DCM-related signalling pathways through various protein factors modulation, which includes RyR-2, TGF-β, IGF-1R, NF-κB and Nrf-2 and also immunological regulation of cardiomyocytes. There has also been a discussion of diagnostic and therapeutic management of various miRNAs in the management of DCM with recent clinical trials on diabetes and cardiovascular disorder with miRNA candidates and concluded with the future perspective of miRNAs as new novel theranostic tools in the emerging field of diagnostic and therapeutic management.

## Graphical Abstract

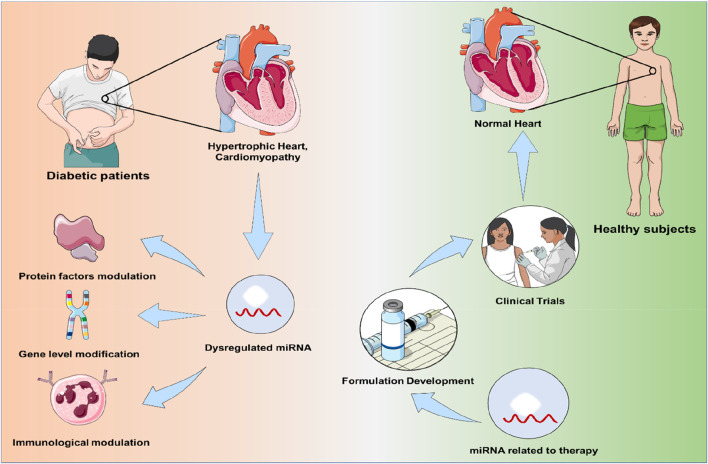

## Introduction

Cardiomyopathy, characterized by structural and functional abnormalities of the myocardium, is a significant contributor to heart failure (HF), a leading cause of morbidity and mortality worldwide. Among its many etiologies, diabetic cardiomyopathy (DCM) has emerged as a distinct and increasingly prevalent condition in the context of the global diabetes epidemic (Schenke-Layland et al., 2009; Cao et al., 2015; Peters, 2016). As of 2021, diabetes mellitus (DM) affects approximately 540 million adults globally—a figure expected to rise to 783 million by 2045—resulting in 6.7 million deaths and imposing an immense economic burden of nearly $966 billion annually (International Diabetes Federation, 2024a; International Diabetes Federation, 2024b). Importantly, cardiovascular diseases (CVDs) account for over 80% of deaths among diabetic individuals, often culminating in HF (Lelieveld et al., 2019; Paul et al., 2023).

DCM develops independently of hypertension or coronary artery disease and encompasses a complex array of pathophysiological changes. These include myocardial fibrosis, lipotoxicity, mitochondrial dysfunction, dysregulated calcium homeostasis, insulin resistance, oxidative and endoplasmic reticulum stress, and an energy shift from glucose to fatty acid metabolism (Banerjee et al., 2025b). Despite extensive research, the molecular mechanisms driving DCM remain incompletely understood, and current therapies primarily target systemic metabolic abnormalities rather than myocardial-specific processes (Boudina and Abel, 2010; Banerjee et al., 2023). Hyperlipidemia and hyperinsulinemia are known to gradually damage pancreatic cells (Singha et al., 2024), which struggle to meet the heightened metabolic demands imposed by chronic overnutrition and a sedentary lifestyle—ultimately leading to cellular dysfunction and death—and this metabolic imbalance may further contribute to heart failure through a glucose-lipotoxicity loop, where chronic hyperglycemia promotes increased fatty acid absorption and myocardial steatosis (Winhofer et al., 2012; Banerjee et al., 2024). The novel anti-diabetic agent canagliflozin, a sodium-glucose cotransporter 2 (SGLT2) inhibitor, has demonstrated efficacy in reducing cardiovascular mortality and heart failure-related hospitalizations in individuals with type 2 diabetes mellitus (T2DM) and cardiovascular disease (CVD), although its precise mechanisms of action in the context of diabetic cardiomyopathy (DCM) remain still unclear (Packer, 2019).

MicroRNAs (miRNAs) are a class of small, noncoding RNAs approximately 22–25 nucleotides long that regulate gene expression at the post-transcriptional level. They have been increasingly recognized for their significant roles in the pathogenesis of various diseases, including cancer, metabolic disorders, and cardiovascular diseases (Sarkar et al., 2023) (Table 1). In the context of diabetes, accumulating evidence suggests that miRNAs are critically involved in the development of diabetic cardiomyopathy (DCM) (Sar et al., 2023b; Saha et al., 2025; Shaharyar et al., 2025). A recent study identified the dysregulation of 316 miRNAs in the hearts of streptozotocin (STZ)-induced diabetic mice, highlighting the extensive impact of diabetes on cardiac gene regulatory networks. Notably, downregulation of miR-30c and upregulation of BECN1 (Beclin-1)—a key autophagy-related protein—were shown to promote autophagy in both diabetic (db/db) mouse hearts and palmitic acid-treated cardiomyocytes (Chen et al., 2017).

**TABLE 1:** Representative microRNAs: Biological Functions, Disease associations, and Theranostic potential.

miRNA	Primary biological function	Disease associations	Diagnostic potential	Therapeutic potential	References
miR-1	Cardiac muscle differentiation, apoptosis regulation	Myocardial infarction, arrhythmias, HF	Biomarker for cardiac injury	Target for reducing cardiac apoptosis	Shan et al. (2010)
miR-21	Fibrosis regulation, anti-apoptosis, inflammation control	Fibrosis (lung, liver, kidney), cancers, CVD	Biomarker for fibrosis and cancer progression	Antifibrotic and anticancer therapeutic target	Thum et al. (2008)
miR-126	Angiogenesis, vascular homeostasis	Atherosclerosis, T2DM, ischemic heart disease	Biomarker for vascular dysfunction	Endothelial repair and pro-angiogenic therapy	Zampetaki et al. (2010)
miR-155	Immune modulation, inflammation mediator	Autoimmune diseases (RA, SLE), cancers, CVD	Biomarker for systemic inflammation	Immunomodulation and anti-inflammatory therapy	O’Connell et al. (2007)
miR-34a	Cell cycle arrest, apoptosis, senescence	Aging, cancer, diabetic cardiomyopathy, neurodegeneration	Biomarker for aging and fibrotic remodeling	Senolytic and anti-apoptotic therapy	Boon et al. (2013)
miR-133a	Anti-hypertrophic, myocyte survival	CH, HF	Cardiac stress biomarker	Anti-hypertrophic therapy	Care et al. (2007)
miR-210	Hypoxia response, mitochondrial metabolism	Ischemic stroke, myocardial infarction, cancer	Hypoxia biomarker	Protects against ischemia-reperfusion injury	Chan et al. (2009)
miR-144	Redox regulation via Nrf2, antioxidant defense	T2DM, metabolic syndrome, CVD	Oxidative stress biomarker	Antioxidant and metabolic therapy	Yu et al. (2015)
miR-378 -3p	Lipid metabolism, mitochondrial function	Obesity, metabolic syndrome, NAFLD	Biomarker for lipid dysregulation	Lipid metabolism modulator	Machado et al. (2020)
miR-29	Fibrosis regulation via ECM proteins	Liver fibrosis, pulmonary fibrosis, renal fibrosis	Non-invasive biomarker for organ fibrosis	Anti-fibrotic therapeutic agent	Van Rooij et al. (2008)
miR-223	Inflammation, myeloid differentiation	T2DM, metabolic syndrome, cancers	Biomarker for immune dysregulation	Inflammation control and metabolic therapy	Fazi et al. (2005)
miR-192	TGF-β signalling, fibrosis modulation	Diabetic nephropathy, kidney fibrosis	Early biomarker for diabetic nephropathy	Antifibrotic agent for kidney disease	Kato et al. (2007)
miR-122	Lipid metabolism, liver function	NAFLD, hepatitis C, hepatocellular carcinoma	Liver function biomarker	Therapeutic target for NAFLD and HCV	Esau et al. (2006)
miR-200	EMT regulation, fibrosis, tumor metastasis	Cancer metastasis, kidney fibrosis, pulmonary fibrosis	Biomarker for tumor invasion and fibrosis	EMT modulation therapy	Korpal et al. (2008)
miR-375	Insulin secretion regulation	T2DM	Biomarker for beta-cell dysfunction	Target for beta-cell preservation and insulin modulation	Poy et al. (2004)

Importantly, these miRNA alterations persisted despite glycemic control, suggesting that hyperglycemia-induced changes in miRNA expression may drive sustained cardiac dysfunction independently of blood glucose levels (Costantino et al., 2016). Mechanistically, miRNAs typically function in the cytoplasm but also exert regulatory roles within the nucleus and mitochondria, primarily acting as post-transcriptional repressors of gene expression (Moshkovich et al., 2011; Li et al., 2016). Although numerous miRNAs have been implicated in disease processes, the specific roles of individual miRNAs in the pathogenesis of DCM remain inadequately understood (Sar et al., 2023a; Sar et al., 2025; Banerjee et al., 2025a). Unraveling these mechanisms could provide novel insights into disease progression and identify potential therapeutic targets for managing diabetic heart complications.

## Molecular pathophysiology of DCM

In sustained hyperglycaemia, when blood glucose levels are not regulated for a prolonged period, diabetic hearts may irreversibly induce the expression of certain regulatory miRNAs. This suggests that these epigenetic regulators may play significant roles in the pathogenesis of cardiac dysfunction in diabetes. It occurs through a series of molecular pathways, which are ultimately reflected as reasons for cardiac cell death, fibrosis, and hypertrophic cardiomyopathy.

However, cardiovascular remodelling and dysfunction are caused by various pathophysiological processes, including increased release of inflammatory cytokines (e.g., NF-κB, IL-6, IL-1, and TNF-α), fatty acid dysregulation, oxidative stress, hyperglycemia-related advanced glycation end products (AGEs) accumulation derived microvascular damage, calcium balance dysregulation, autoimmunity, insulin resistance, and hyperinsulinemia, which have been further discussed below.

## Abnormal free fatty acid metabolism

The development of DCM is also accompanied by an increase in the capacity of myocyte sarcolemma free fatty acid (FFA) transporters and an increase in FFA release from adipose tissue (Guo and Guo, 2017). It is important to highlight that DCM has multiple causes, including toxic lipid accumulation and toxic glucose metabolites. This is a clinical characteristic of diabetic cardiomyopathy and an independent predictor of diastolic dysfunction.

Fatty acid oxidation, *de novo* lipogenesis (DNL), import of FFA, and export of triglyceride-rich, very low-density lipoproteins (LDL) are all regulated by Forkhead box, subclass O (FOXOs), which are essential for triglyceride homeostasis. FOXOs regulate DNL by suppressing the transcriptional expression of the lipogenic master regulator SREBP 1 and activating adipose triacylglycerol lipase, hormone-sensitive lipase, lipoprotein lipase, and carnitine palmitoyltransferase 1 gene for lipolysis and fatty acid oxidation. FOXO1 has demonstrated the upregulation of fatty acid transporters, including Cluster of Differentiation 36 (CD36) (Dong, 2017).

Diabetic hearts have higher levels of CD36, a protein and transporter that is mostly membrane-localized and stimulates FFA uptake in endosomal and sarcolemma membranes (Lee et al., 2017). CD36 also has another significant role in stimulating FFA assimilation into myocardium cells through AMP-activated protein kinase (AMPK). The phenomenon of cardiomyocyte FFA absorption, getting reduced by 70% in CD36 knockout (KO) mice, supports the hypothesis (Shu H. et al., 2022). Several studies have suggested a negative correlation of CD36 with Peroxisome proliferator activated receptor (PPAR)α deficient mice models (Watanabe et al., 2000). PPAR isoforms were found to be expressed in the cardiomyocytes and are essential for the metabolism of lipids in the myocardium. PPARα regulates the transport and oxidation of fatty acids and ketogenesis to facilitate the adaptive response to fasting. Moreover, PPARγ is a key transcriptional regulator of adipogenesis, promoting lipid uptake and storage in adipose tissue, which in turn improves systemic glucose metabolism and insulin sensitivity. In addition, it is believed also to have antihypertrophic and antiatherosclerotic effects (Takano et al., 2000; Gross et al., 2017).

However, ER stress related to lipids is exacerbated by diacylglycerol. A high-fat diet (HFD) increases membrane diacylglycerol levels, activates protein kinase C (PKC), causes IR, and decreases NO generation (Jornayvaz et al., 2011; Cantley et al., 2013). Moreover, some literatures suggest that the generation of ROS stress is one of the mechanisms by which FFA oxidation and AGE accumulation precipitate DCM (Chen et al., 2022; Khalid et al., 2022). So, oxidative stress is also an important factor to promot DCM pathogenesis.

## Oxidative stress-mediated pathogenesis of DCM

Clinically, oxidative stress contributes to the progression of DCM toward heart failure and represents a valuable target for early diagnosis and therapeutic intervention, with antioxidants and ROS-modulating agents showing potential for improving cardiac outcomes in diabetic patients. AGE, which is one of the important factors to promote ROS in diabetes, combined with receptor advanced glycation end product (RAGE) acts in two ways. Firstly, it causes inactivation of thioredoxin by increased nitration and thioredoxin interacting protein expression, resulting in loss of the anti-apoptotic and anti-oxidative function of the cardiomyocytes. In another way, the AGE-RAGE complex can stimulate nitric oxide production, which generates ROS combined with nitrous radicles to make reactive nitrogen species (RNS) (De Geest and Mishra, 2022; Di Meo et al., 2016). These free radicals damage cells and augment oxidative stress, which stimulates the formation of poly ADP ribose polymerase (PARP), a DNA-repairing enzyme that triggers necrosis by lowering the amount of ATP in cells to make up for damaged DNA (Cantó et al., 2013). Researchers suggested that activation of PARP is also responsible for the elevation of TGF-β, which leads to collagen deposition and perivascular fibrosis. ROS or RNS also activates NF-κB mediated signalling, leading to the formation of the β-myosin heavy chain, resulting in contractile dysfunction and interventricular septum hypertrophy (Moris et al., 2017).

However, this ROS leads to the production of Angiotensin II (AT II) by excessive renin-angiotensin-aldosterone system (RAAS) stimulation. It also induces endoplasmic reticulum (ER) stress by misfolding ER proteins, which may play a role in the pathogenesis of DCM (Nurun Nabi and Ebihara, 2021). Moreover, in DCM, increased ROS generation conveys a strong proinflammatory signal and damages cardiac tissue. Furthermore, the hyperglycemia associated with T2DM causes increased mitochondrial respiration in endothelial cells increasing ROS generation and OS.

## Inflammation mediated DCM

Inflammation is crucial to the pathogenesis of DCM (Frantz et al., 2018; De Angelis et al., 2019). Excess glucose or FA in the heart stimulates NF-κB, a protein complex that regulates DNA transcription, and thereby induces proinflammatory cytokines (IL-6, pro-IL-18, pro-IL-1β, and TNF-α) and NLRP3 inflammasome formation. In the same way, AGE/RAGE signalling promotes NF-κB activation and mediates an inflammatory reaction by heterodimerizing with toll-like receptor-4 (TLR4), resulting in the production of NLRP3, pro-IL-1β, and pro-IL-18 (Jia et al., 2018). The pathogenesis of HF in diabetes is significantly influenced by the activation of the NLRP3 inflammasome, which leads to the proliferation and infiltration of inflammatory cells (AlSaeed et al., 2024). In another study, it was observed that DPP-4 inhibitors reduced NLRP3 inflammasome-mediated inflammatory effects in macrophages by inhibiting the PKC pathway, which thereby reduced ROS generation and NLRP3 activities (Dai et al., 2014).

The stimulation of several serine/threonine kinases, including PKCs, IKK, and c-Jun NH2-terminal kinase (JNK), is linked to increased oxidative stress (Yung and Giacca, 2020). Their activation causes phosphorylation at the serin moiety of insulin receptor substrates (IRS-1) and reduction of PI3K/Akt, leading to inhibition of the dislocation of Glucose transporter type 4 (GLUT4) and glucose transport (Stuart et al., 2014). These processes influence pro-inflammatory cytokines, insulin signalling and transcription, resulting in IR. NF-κB and activator protein (AP)-1 control the transcription of pro-inflammatory genes like *IL-6*, *IL-1*, and *TNF-*α, activated by ROS. Additionally, NF-κB promotes the production of adhesion molecules, including ICAM, VCAM, and E-selectin, which aid in the pathophysiology of the vascular system (Masenga et al., 2023). However, it is also important to note that PPAR upregulation facilitated with CD36 expression in a diabetic condition also triggers inflammatory signallings (AlSaeed et al., 2024).

However, TNF-α and IL1β have been demonstrated to suppress the expression of Ca^2+^-regulating genes (SERCA2a and Ca^2+^ release channel), leading to a negative ionotropic effect due to alteration in [Ca^2+^]_i_ homeostasis in adult cardiomyocytes. Abnormal sarcoplasmic reticulum Ca^2+^ release promotes myocardial remodelling (hypertrophy, significant fibrosis, ventricular dilatation, and pump failure), which leads to heart failure owing to pressure overload. These findings show that inflammation-induced Ca^2+^ imbalance can lead to cardiac remodelling.

## Dysregulation of calcium homeostasis

Calcium homeostasis has a significant role in cardiac contractile and relaxation function via Ca^2+^-mediated transporters. Clinically, calcium dysregulation exacerbates diastolic and systolic dysfunction in diabetic hearts, making it a potential target for early diagnosis and therapeutic strategies to prevent heart failure progression in diabetic patients. Depolarization occurs when the action potential reaches cardiac tissue, activating the Ca^2+^ voltage-gated channel, which leads to an influx of Ca^2+^ ions (Eisner et al., 2017). These ions induce Ca^2+^ efflux from the sarcoplasmic reticulum (SR) by phosphorylation through activation of ryanodine receptor (RyR) type 2, responsible for actin-myosin contraction. After these, Ca^2+^ is uptaken by stimulation of several channels like sarcolemmal Ca^2+^ ATPase Channel (SERCA), Na^+^-Ca^2+^exchanger (NCX) and plasmalemma Ca^2+^ ATPase channel. In the hyperglycaemic heart of the HFD mouse model of type-2 DM, it was illustrated that elevation of intercellular Ca^2+^ concentration downregulates SERCA2 activity, and this high concentration Ca^2+^ upregulates RyR2, which ultimately results in contractile dysfunction, leads to ventricular dysfunction which is a common cause of cardiomyopathy (Foti et al., 2020). DCM and HF are caused by RyR2 phosphorylation at pathological levels. According to studies on human cardiac tissue, RyR2 phosphorylation and mutations have been linked to deadly ventricular arrhythmias, atrial fibrillation, malfunction of the SA and AV nodes, atrial stalemate, dilated cardiomyopathy, HF, and sudden cardiac death (Foti et al., 2020; Tester et al., 2020). Increased oxidative stress brought on by the production of ROS, such as superoxide anions, hydrogen peroxide, and hydroxyl radicals, is associated with diabetic problems. These substances can interact with reactive cysteines, leading to RyR2 malfunction. Different oxidizing substances have been demonstrated to enhance RyR2 by oxidizing sulfhydryl (Nikolajević Starčević et al., 2019; De Geest and Mishra, 2022).

In summary, RyR receptors are crucial for understanding the occurrence of DCM by calcium dysregulation. Their dysfunction, ultimately, has a significant role in cardiac problems, which is a characteristic of this pathogenic condition.

## Biogenesis of miRNA

**FIGURE 1:**
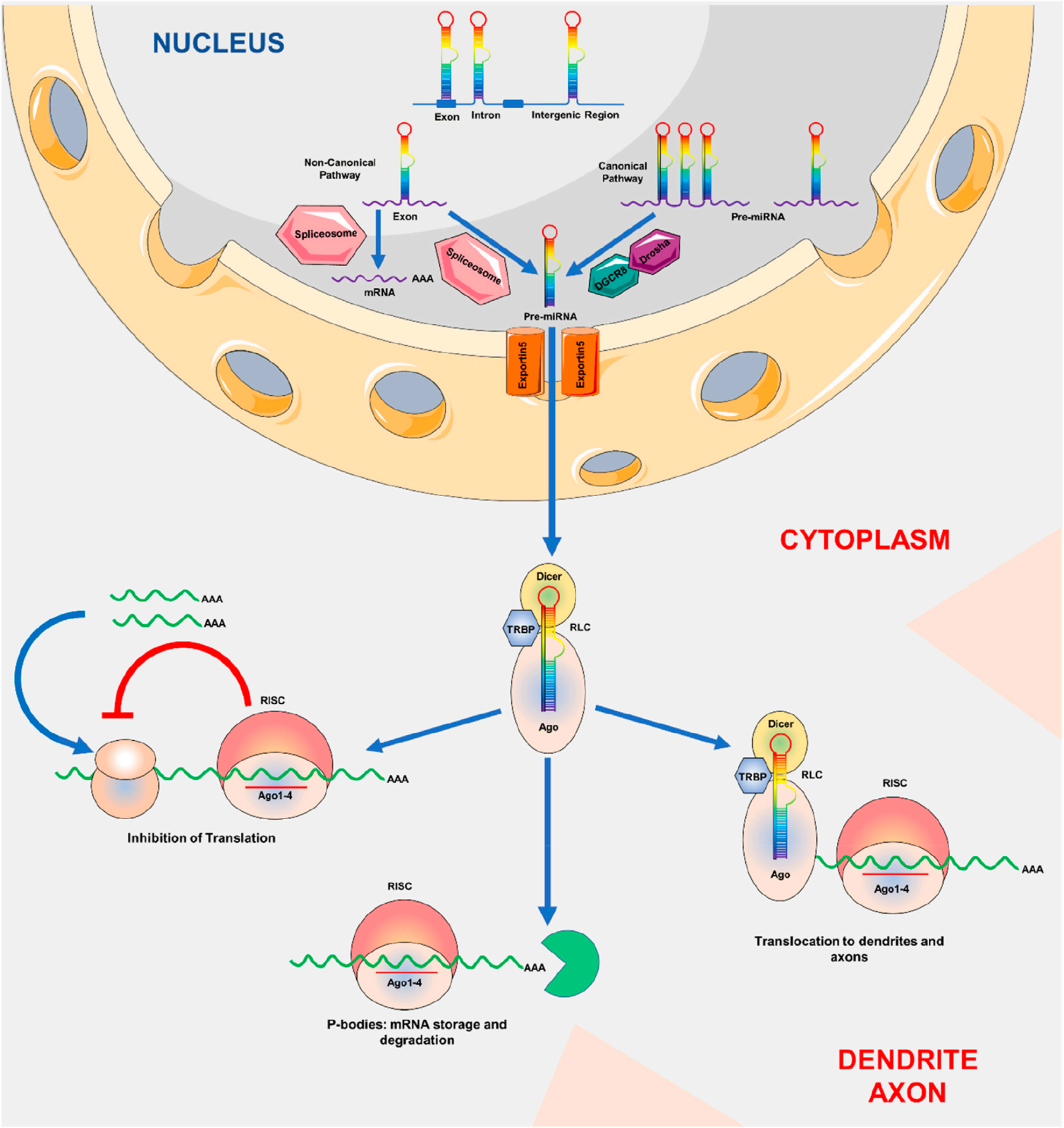
Biogenesis of miRNA: MicroRNA production. The genome is used to transcribe pri-miRNAs, which Drosha and Pasha then convert into pre-miRNAs with a length of around 70 nt. RanGTP/exportin-5 is required for the export of pre-miRNAs from the cell nucleus to the cytoplasm. Pre-miRNAs are broken up by Dicer close to the hairpin loops and transferred to the miRISC complex. It has been established that miRNAs that fully complement target mRNA either cause mRNA to be degraded or separate it in P bodies for translation suppression.

The biogenesis of miRNA broadly follows two routes e.g., canonical biogenetic routes and non-canonical biogenetic routes (Figure 1). However, the canonical biogenesis route is the main mechanism for processing miRNAs. Through this pathway, ribonuclease III enzyme Drosha and DiGeorge Syndrome Critical Region 8 (DGCR8) protein, which binds to RNA, transforms pre-miRNAs from their genes into pri-miRNAs (O’Brien et al., 2018). While Drosha cleaves the pri-miRNA duplex at the base of the distinctive miRNA hairpin structure, DGCR8 detects an N6-methyl-adenylated GGAC and other motifs within the pri-miRNA and develops pre-miRNA with 2 nt 3′overhang (O’Brien et al., 2018). Pre-miRNAs are synthesized, and an exportin 5 (XPO5)/RanGTP complex brings them into the cytoplasm, which is then processed by the RNase III endonuclease Dicer. The terminal loop is eliminated during this procedure, creating a mature miRNA duplex (Zhang and Zeng, 2010). The directionality of the miRNA strand sets the naming scheme of the mature miRNA form. The pre-miRNA hairpin has two ends, the 5′end and the 3′end, corresponding to the 5p and 3p strands. The Argonaute (AGO) family of proteins (AGO1-4 in humans) can ATP-dependently load both strands from the mature miRNA duplex (Yoda et al., 2010). Depending on the percentage of AGO in a cell or cellular environment, the 5p or 3p strand changes significantly for any particular miRNA, varying from about equal proportions to predominately one or the other (Meijer et al., 2014). Based on thermodynamic stability, 5p or 3p strand is selected at the 5′ends of the miRNA duplexor, a 5′U strand, at nucleotide position 1 (O’Brien et al., 2018). The strand with lesser 5′stability or 5′uracil is often loaded into AGO preferentially and is referred to as the guide strand. Depending on the level of complementarity, the passenger strand, also known as the unloaded strand, will be unwound from the guide strand using various methods. Argonaute RISC Catalytic Component 2 (AGO2) cleaves the passenger strands of miRNA that do not have any mismatches, and then the cellular machinery degrades them, which can result in a severe strand bias. Without AGO2 loading, miRNA duplexes with central mismatches degrade and unwind passively (Ha and Kim, 2014).

Few non-canonical miRNA biogenesis mechanisms have been discovered to date. These routes primarily incorporate Drosha, Dicer, exportin 5, and AGO2 with other components from the canonical pathway. Two types of non-canonical miRNA biogenesis mechanisms exist Drosha/DGCR8-and Dicer-dependent. Pre-miRNAs produced in the Drosha/DGCR8-un reliant pathway mimic Dicer substrates (Yang and Lai, 2011).

Furthermore, miRNA can regulate various other epigenetic modifications, including changes in the amounts of histone H2A mRNA, histone deacetylase (HDAC), and DNA methyltransferases (DNMT). This ultimately leads to the modulation of gene modification and expression (Yang et al., 2010). Under conditions of elevated glucose levels, signals mediated by miRNA can be transferred to other cells or tissues (Cheloufi et al., 2010). The amount of circulating miRNAs in the blood can be changed at different stages of DCM. Collectively, miRNA could potentially serve as a diagnostic, prognostic, and therapeutic tool in the prevention and treatment of DCM (Cao et al., 2020).

## Genetic roles to microRNA with regional impact in progression of DCM

Advancements in sequencing technology have progressively unveiled the extensive regulation of miRNAs during the development of DCM. Distinct miRNA expression profiles have been reported at various phases of DCM.

miRNAs regulate gene expression by binding to distinct genes’ 3′UTRs, meaning that they may be involved in a variety of DCM pathological processes. miRNA-30c expression levels were reduced in db/db mice; however, its localised overexpression at the cardiac location improved lipid accumulation, ROS production, and cardiomyocyte death. Furthermore, miRNA-30c affects apoptosis-related genes such as *beclin1*, *p53*, and *p21*, which prevent diabetes-induced cardiomyocyte apoptosis. The same downregulated miRNA133a found in diabetic hearts has a role in cardiac remodelling. In conjunction with *COL1A1*, *ERK1/2*, and *Smad2, miRNA-133a inhibits collagen synthesis in* cardiac fibrosis (Ma et al., 2024).

Numerous studies support the preventive role of miR-21 in cardiovascular disorders. miR-21 enhances fibrosis and cell death in cardiomyocytes; additionally, the hypoglycemic agent vildagliptin exhibits both hypoglycemic and cardioprotective effects via the miR-21/SPRY1/ERK/mammalian target of rapamycin pathway. The p38/mitogen-activated protein kinase (MAPK) signalling pathway is significantly elevated in diabetes and plays a crucial role in several pathogenic processes of DCM, including oxidative stress, apoptosis, and ventricular remodelling (Li H. et al., 2022). HG-induced miR-21 overexpression promotes the downstream p38/MAPK pathway, hence contributing to ventricular remodelling in DCM (Ma et al., 2024).

Moreover, miR-320 is exclusively expressed in the cardiomyocytes of DCM animals and can be identified in the plasma prior to any impairment in ventricular diastolic function. In contrast to cytoplasmic miRNAs, nuclear miR-320 can bind to the promoter of the *CD36* gene, which encodes a fatty acid receptor, resulting in its expression (Mittal et al., 2021).

However, numerous miRNAs have been identified as regulators of cardiac fibrosis and cardiac hypertrophy in DCM. For example, miRNA-221 was demonstrated to be significantly increased in the heart tissue of diabetic mice (Ucar et al., 2012). miRNA-212 was identified as a regulator of ventricular hypertrophy by its direct modulation of *Foxo3*. Recent reports indicate that the inhibition of miR-199a resulted in the reversal of ventricular hypertrophy by restoring mitochondrial fatty acid oxidation via the modulation of PGC-1α (Yan et al., 2021). Levels of miRNA-30a, miRNA-1, and miRNA-29b were observed to be downregulated in the diabetic heart (Costantino et al., 2016).

Moreover, it is also important to note that regional differences in gene expression play a crucial role in the progression of DCM. Due to genetic variances, cardiovascular illness in Asian populations differs from that in Caucasians. A pooled study from India found that the course of CAD begins earlier in some Asian groups than in Caucasian people, and the mortality rate due to acute macrovascular incidents is significantly higher. The primary reason could be that Asian individuals exhibit higher levels of insulin and blood lipids after the onset of T2DM than Caucasian individuals, which likely causes more severe insulin resistance and endothelial disorders (Unnikrishnan et al., 2016). Because the United States has a large immigrant population, studies can analyse genetic differences in diabetes prevalence within the same region. A study of 49,574 patients showed that between 1997 and 2004, racial variations in diabetes prevalence were detected among different BMI groups, with the highest racial inequalities within the normal BMI groups, and this gap is showing a growing tendency of DCM (Liu R. et al., 2022). However, many studies have addressed the incidence of diabetes among various ethnic groups in China. This is much less common in Tibetans than in Han Chinese. Aside from the previously discussed variations in economic and lifestyle factors, Tibetans are more prone to develop diabetes (Stewart et al., 2016).

However, the global risk of diabetic cardiovascular disease has gradually declined as medical care in most wealthy countries has improved. This may be attributable to improved blood sugar control in diabetic patients, it could also be connected to a drop in the incidence of CVD itself.

## miRNA, as a biomarker in mechanistic regulation and expression of various protein factors responsible for DCM

**FIGURE 2:**
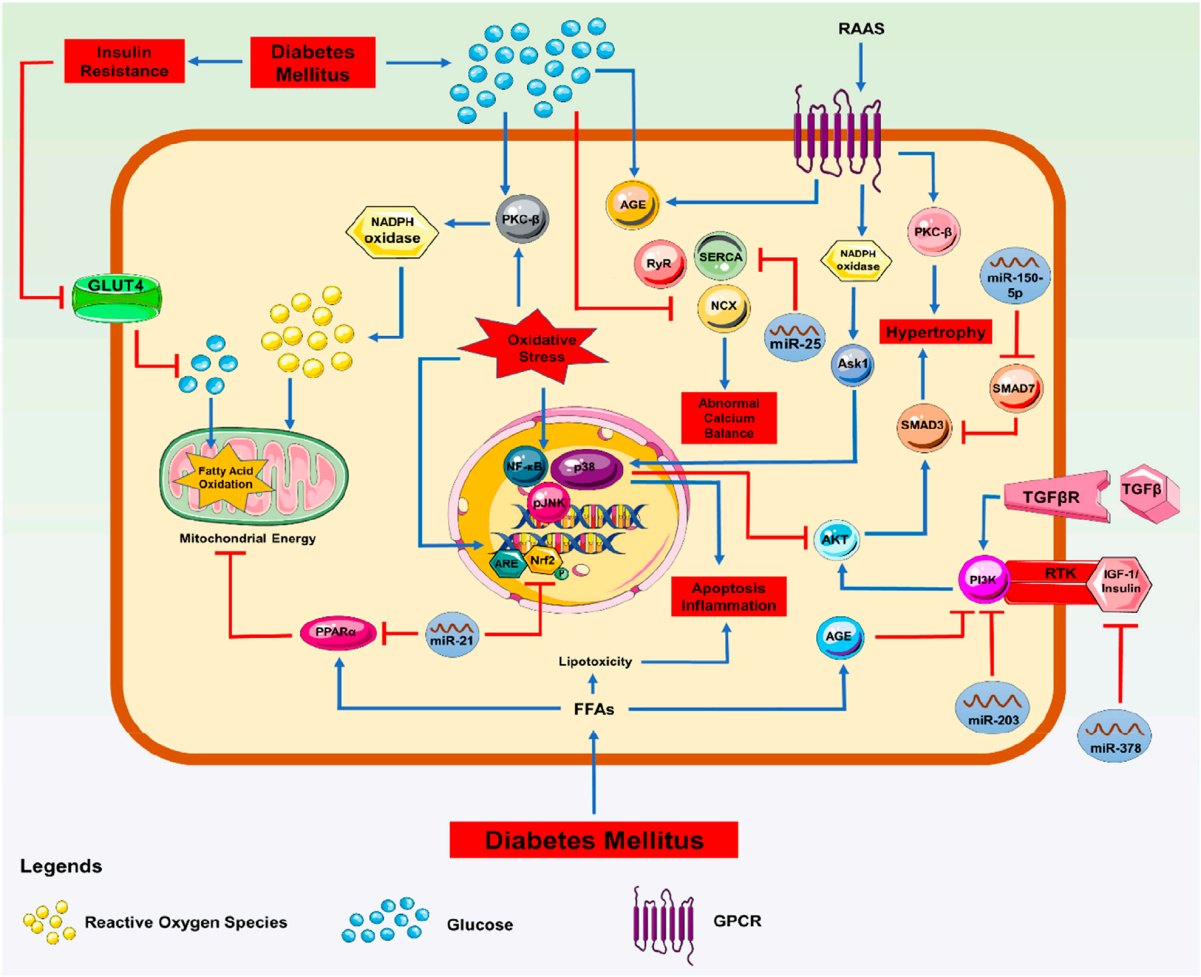
Molecular Signalling with miRNA: Signalling pathway involved in the progression of DCM with miRNA regulations.

In DCM, miRs were discovered to be dysregulated and selectively expressed in various biomolecular signalling pathways. In particular, miR-221 and miR-212, which were associated with the autophagic response and hypertrophy in these people, were markedly overexpressed. Similarly, the upregulation of miR-451, miR-1/206, miR-195, miR-378, miR-320, and miR-34a was associated with cardiomyocyte death (Cao et al., 2020). Several miRNAs, including miR-378, 133a, 1, and 373, were also downregulated in situations with growing hypertrophy and increasing oxidative stress (Klimczak-Tomaniak et al., 2022; Kolodziej et al., 2022; Jayavaram et al., 2023). Based on miRNA discoveries and changes over time, these observations support the involvement of these regulators in phenotypic diagnosis, DCM identification, and the potential identification of biomarkers. According to the preliminary results, it is possible that signal transduction failure contributes to the development of DCM, and that the upregulation or downregulation of miRNA is related to specific molecular pathways (Figure 2). Additionally, the advancement of DCM despite the return to normal glycaemic levels may be explained by the continuous modification of miRs brought on by high blood glucose levels, as demonstrated in diabetic hearts, which exhibit hyperglycaemic memory. Therefore, a few protein factors that regulate miRNA expressions in managing DCM have been briefly discussed below.

## TGF-β

Various cells and organs release a multifunctional cytokine called TGF-β, which exerts diverse stimuli, including hyperglycaemia (Dobaczewski et al., 2011). In the TGF-β-induced cardiac fibroblast model, it was found that TGF-β stimulation triggers fibroblast activation, including fibroblast-to-myofibroblast differentiation, collagen synthesis, and ECM protein deposition via the Smad2/3 pathways (Watkins et al., 2012; Khalil et al., 2017; Shu J. et al., 2022). Additionally, it has been noted that TGF-β released by cardiomyocytes triggers hypertrophic responses via an autocrine mechanism (Wu et al., 2018). Transgenic mice overexpressing TGF-β develop CH and interstitial fibrosis (Wermuth and Jimenez, 2018). However, pressure-overload-induced CH and fibrosis are prevented by cardiomyocyte-specific TGF-β receptor deletion (Koitabashi et al., 2011). These results suggest that TGF-β may play a crucial role in the aetiology of heart fibrosis and hypertrophy. A study revealed that mice developed myocardial fibrosis through a significant upregulation of collagen I and III, as well as TGF-β1 mRNA. These had a substantial association with cardiomyocyte hypertrophy (Hu et al., 2021).

The PI3K/Akt pathway is a crucial component in TGF-β signalling, which also regulates Smad3 activity in some cell types. TGF-β1 stimulates the PI3K pathway, which in turn enhances the transcriptional activity of Smad3, leading to increased expression of collagen I. According to a study in the murine model of DCM, miR-203 and the PI3K/Akt pathway were found to be involved. A downregulated miR-203 can affect the PI3K/Akt pathway in diabetic mice by targeting PIK3CA. A correlation was found between miR-203-mediated suppression of the PI3K/Akt pathway in cardiomyocytes and the reduction of CH and myocardial cell death (Yang et al., 2019).

On the other hand, like PI3K/Akt, Smad7 is also a key component in the TGF-β1 signalling cascade. It inhibits the phosphorylation of receptor-regulated Smads, including Smad3, hence blocking the TGF-β1 signalling pathway. Significant evidence has demonstrated that downregulating the expression of Smad7 considerably worsens myocardial fibrosis (Luo et al., 2023).

High glucose (HG) levels elevated the concentration of miR-150-5p, which directly interacted with Smad7 and downregulated the expression of Smad7 in cardiomyocytes. Blocking miR-150-5p improved the damage caused by HG levels in the cardiomyocytes by deactivating the TGF-β1/Smad signalling pathway, which leads to cardiac fibrosis. The inhibition of miR-150-5p resulted in an increase in Smad7 levels compared to treatment with HG. The transfection of AMO-150-5p, anti-miRNA oligonucleotides, resulted in a noteworthy reduction in the levels of TGF-β1, p-Smad2, and p-Smad3. This indicates that the suppression of miR-150-5p deactivated the TGF-β1/Smad signalling pathway by directly targeting Smad7 (Che et al., 2020).

The miR-34a plays a significant role in regulating the functions of vascular smooth muscle cells and inhibiting the excessive growth of neointima, suggesting its potential use in treating vascular disorders. The miR-34a molecule improves the condition of myocardial fibrosis in DCM by decreasing the formation of type I collagen, reducing cell viability and migration, and enhancing cell function (Hua et al., 2022). This is achieved by targeting the pin-1 protein, which is found in mouse cardiac tissues via immunohistochemistry labelling (Zhang X. et al., 2021). It was found that miR-142-3p directly regulates TGF-β1 when exposed to HG levels. In human aortic endothelium cells (HAECs), the decrease of miR-142-3p is expressed with time and dose. miR-142-3p has the ability to target TGF-β1, specifically by binding to a location in the 3′-UTR of TGF-β1. The overexpression of miR-142-3p suppressed the production of TGF-β1 and decreased the phosphorylation of Smad2/3 in HAECs treated with HG (Zhu et al., 2018). This is how miR-142-3p prevents EMT from occurring via lowering HG levels by inhibiting the TGF-β1/Smad pathway. The miR-142-3p/TGF-β1/Smad axis could be a potential attack point for DCM treatment (Zhu et al., 2018). The miR-9 negatively regulates the signalling pathways of TGF-β and inflammation, thereby governing the EMT process, which is controlled by epigenetic mechanisms and involves the transformation of endothelial cells (Wang et al., 2023). It is noted that in MS-1 and 2H11 cells, it is investigated that TGF-β is a potent inducer of EMT. Additionally, it triggered EMT in human coronary endothelial cells and led to cardiac fibrosis, ultimately causing heart hypertrophy (Ma et al., 2021). Elevated glucose levels has been found to facilitate endothelial deterioration by inducing EMT through the suppression of miR-9 (Wang et al., 2023).

## Nrf-2

Among the cap’n'collar family of transcription factors, a crucial modulator of cellular detoxification pathways and the state of redox is NFE2-related factor 2 (Nrf2) (Hammad et al., 2023). Kelch-like ECH-associated protein 1 (KEAP1) may initiate the rapid ubiquitination and subsequent degradation of Nrf2 (Dodson et al., 2022). When cells are exposed to oxidative stress, Nrf2 is released from KEAP1. In the nucleus, the antioxidant-responsive elements (AREs) in the genes encoding NADPH quinone oxidoreductase (NQO1), glutathione S-transferase, and Heme oxygenase-1 (HO-1) are bound by Nrf2 (McMahon et al., 2003; De Haan, 2011). Since numerous studies have summarised that HG induces ROS and RNS in cultured cardiovascular and renal cells, it has been investigated whether HG could increase Nrf2 expression and its downstream gene expression (Zhang et al., 2022; Adoga et al., 2022) demonstrated the decrease in Nrf2 mRNA expression in the cardiomyocytes of STZ-induced diabetic rats, indicating that Nrf2 expression was downregulated in the heart of diabetic rats. The decrease in Nrf2 expression suggests a decline in antioxidant enzymes and an increase in lipid peroxidation, indicating oxidative stress, which may lead to inflammation and reduced endothelial function. Another *in vitro* study also showed that in primary cardiomyocytes and H9C2 cells, 24-h treatment with glucose boosted the expression of Nrf2 mRNA. Such HG exposure caused the overexpression of NQO1, a prototype of an Nrf2-regulated chemical detoxification gene. HG treatment considerably raised the Nrf2 of the cells. This suggests that glucose did indeed boost Nrf2 protein levels and nuclear accumulation (He et al., 2009). It was also noted that autopsied hearts of diabetic and normal people were used to acquire tissue sections of the left ventricles. Compared to the non-diabetic heart, Nrf2 expression was dramatically downregulated in the nuclei.

In DCM animal models, increased Nrf2 provides antioxidant benefits, protecting cardiomyocytes against OS-induced damage. Agonists that may influence Nrf2-associated epigenetic mechanisms include methylation of the nfe2l2 promoter and miR-144, miR-155, and miR-503 inhibitors, which increase Nrf2 expression to reduce cellular OS (Peng et al., 2022). Furthermore, multiple studies suggest the possibility of reciprocal regulation between the PPARs and Nrf2 signalling pathways, which mutually promote the expression of both pathways (Reddy and Standiford, 2010; Polvani et al., 2012). Secondary changes in oxidative stress activate Nrf2 through PGC-1α after PPARα activation (Li D. et al., 2017). *LAZ3*, a gene that encodes a protein, functions as a transcriptional repressor and controls inflammation by impeding the NF-κB signalling (Gao et al., 2018). This pathway is also connected to Nrf2 and PPARα activation. Researchers detected a lower level of *LAZ3* in the cardiomyocytes of diabetic murine models (Barish et al., 2010).

An experiment found that overexpressing LAZ3 in the heart increased PPARα expression and Nrf2 activation. This study identified one of the most striking phenotypes: LAZ3 activates PPARa-Nrf2 signalling, which affects glucose and lipid metabolism. These pathways may explain the LAZ3-mediated anti-inflammatory and anti-oxidative activities in the DCM heart, as both Nrf2 knockdown and PPARα agonists can reverse the effects of LAZ3 overexpression or silencing (Gao et al., 2018). Another study found that overexpressing LAZ3 (by gene delivery of LAZ3 with Ad-LAZ3) protects the heart from HG and slows the progression of DCM through the miR-21/PPARa/Nrf-2 signalling pathway (Mollajan et al., 2025). Similarly, the expression of miR-21 in cardiomyocytes, which targets PPARα, was found to be inhibited by *the upregulation of LAZ3*. Silencing *LAZ3* results in reduced activation of Nrf2 and PPARα, thereby compromising the cellular response to oxidative stress. Similar to these findings, cardiac miR-21 release has been observed in an unselected cohort group of individuals with non-ischemic cardiomyopathy, including DCM (De Rosa et al., 2018).

Furthermore, in diabetic cardiomyocytes, miR-144 was revealed to be downregulated in its role as an OS regulator. The miR-144 levels were lowered in the diabetic murine models, and under HG circumstances, cardiomyocytes, the incorporation of miR-144 mimic decreased the Nrf2 expression and increased ROS, whilst miR-144 inhibitor increased Nrf2 expression and lowered ROS production (Tao et al., 2020).

Tao et al. (2020) have discovered discrepant findings. Increased diabetic cardiac dysfunction may be associated with miR-503 overexpression in endothelial cells, and the phase II enzyme inducer CPDT may improve Nrf2 activation. This enzyme complex stimulates the production of antioxidant enzymes and plays a critical role in protecting against oxidative stress (Wang et al., 2017). It has been postulated that CPDT may have a similar impact on diabetic cardiac dysregulation, and that downregulation of miR-503 may reduce the development of DCM. Miao et al. studied the association between CPDT and miR-503 in DCM (Miao et al., 2017). It also employed CPDT as a therapeutic agent. Compared to untreated diabetic rats, those given CPDT showed lower expression of miR-503 and higher levels of its target, Nrf2, as well as other detoxifying enzymes, including Malondialdehyde (MDA) and HO-1. According to the findings, CPDT protects against cardiomyopathy by decreasing miR-503 expression and increasing Nrf2 expression, thereby reducing cardiac cell death and slowing the progression of the disease. DM is associated with a change in miRNA abundance in the heart muscle. *In vivo* and *in vitro* models of DCM showed downregulated expression of miR-21 and miR-144 in cardiac tissue, while miR-503 was upregulated. These miRNAs impact the anti-oxidative function and diminish its capacity to avoid the harmful consequences of oxidative stress caused by the increased ROS in DM.

## IGF-1R

The highly conserved insulin-like growth factor 1 (IGF-1) signalling pathway regulates growth, development, and metabolism. It regulates various cellular functions, including cell division, energy metabolism, and glucose homeostasis (Chen et al., 2010; Smith-Vikos and Slack, 2012). IGF-1 expression in VSMCs and endothelial cells (ECs) has been linked to insulin resistance, glucose intolerance, an increased risk of T2DM, and cardiovascular morbidity and mortality. It also correlates independently with coronary microvascular dysfunction, indicating that this growth factor plays a critical role in increasing cardiac risk. A recent case-control study of over 4,000 individuals suggested that the IGF-1 signalling pathway contributes to the risk of stroke incidence (Macvanin M. et al., 2023). The IGF-1 signalling cascade comprises transmembrane receptors, ligands, and IGF binding proteins (IGFBPs), which regulate effector molecules (Baxter, 2023). IGF-1 interacts with the heterotetrameric transmembrane receptor tyrosine kinase known as IGF-1 receptor (IGF-1R). Two pro-survival signalling pathways are activated by IGF-1R activation, which recruits and phosphorylates several adaptor proteins, including IRSs and Shc. While phosphorylation of Shc activates the extracellular signal-regulated kinase (ERK)/Mitogen-activated protein kinase (MAPK), RAS, rapidly accelerated fibrosarcoma (RAF) signalling cascades, activates the phosphoinositol 3-kinase (PI3K)-PDK1-AKT signalling network. Additionally, to other effects, AKT activation via PDK1 or mTORC2 phosphorylation at Ser473 or Thr308 leads to suppression of cell death and boosts gene expression that supports cell survival.

The role of IGF-1R and IR signalling in cardiac ageing has been revealed through cardiac-specific amplification of the IGF-insulin receptor, which was discovered to accelerate cardiac ageing in *Drosophila* (Abel, 2021). When cardiac-restricted IGF-1-overexpressing mice were employed in mammals, cardiac stem cell activity was preserved, protecting from cardiac senescence (Torella et al., 2004; Li et al., 2007). However, the high plasma IGF-1 levels in these transgenic mice made it challenging to determine the systemic and cardiac effects of IGF-1 (Reiss et al., 1996). IGF-1 enhances cardiac contractility and reduces cardiomyocyte death in experimental models subjected to ischemic conditions (Duerr et al., 1995; Lee et al., 1999; Opgaard and Wang, 2005). Research conducted also identified it as a plausible risk factor contributing to the onset of Congestive Heart Failure (CHF) (Vasan et al., 2003).

Postnatal cardiac remodelling has been reported to decrease IGF-1R levels (Cheng et al., 1995). The function of miRNAs is to reduce IGF-1R levels during the postnatal period, as studied by Knezevic et al. (2012). In the mouse neonatal cardiac tissue (1 week after delivery) and the foetal myocardium (2 weeks’ gestation), the expression levels of several arbitrarily chosen miRNAs were examined. In neonatal cardiomyocytes compared to foetal hearts, miR-378 levels were considerably higher by a factor of more than 10, indicating a significantly abundant miRNA in the heart. Downregulation of miR-378 has a function in cardiac remodelling and the survival of cardiac cells against stress factors by negatively legislating IGF-1R (Yu et al., 2013) as this miRNA is explicitly targeted to IGF-1R 3′UTR, and this upregulation increased apoptotic cell death of cardiomyocytes with decreased IGF-1 signalling action (Jung and Suh, 2015). Another study demonstrated in STZ-induced C57BL6J mice a synergistic activation of the IGF-1R pathway and cell proliferation in miR-378a-deficient HG-treated hiPSC-CMs. Thus, miR-378a overexpression in HG circumstances may be regarded as a compensatory mechanism to diminish pro-hypertrophic pathways (Florczyk-Soluch et al., 2023).

miR-1 also significantly modulates the IGF-1R signalling pathway in cardiac muscle cells, as well as in skeletal muscle (Huang et al., 2011). In particular, evidence from specific studies strongly shows that miR-1 is a regulator and moderator of the diverse effects of IGF-1 on heart muscle (Elia et al., 2009). miR-1 may target IGF-1, as proposed in the past (McCarthy and Esser, 2007). miR-1 repressed the cellular mRNA and protein levels of IGF-1 by directly targeting the binding site within the 3′untranslated region (3′UTR), which subsequently led to a reduction in cell viability. These results suggested that miR-1 negatively regulates IGF-1 expression at the post-transcriptional level (Hu et al., 2013). However, the functional relationship’s biological importance and its role as a component of the automodulatory loop in cardiac and skeletal muscles demonstrate that it is a biological activity. This significance is emphasised by data from human samples showing how the miR-1 and IGF-1 control loop regulates human VH (Elia et al., 2009). miR-30d affects the modulation of IGF-1 via insulin, and extended exposure of MIN6 cells to HG leads to the differential expression of miR-30d. Increased levels of miR-30d in hyperglycaemic cardiomyocytes enhance the production of insulin genes, while suppressing miR-30d reduces the transcription of insulin genes in response to glucose stimulation and modulates IGF-1 mediated DCM (Shantikumar et al., 2012).

## RyR-2 and SERCA2a

The necessity of RyR2 for myocardial contractions, especially rhythmic contraction and myocardial relaxation, has been demonstrated in numerous investigations. In smooth muscle cells, RyR1 exhibits subordinate expression, influencing artery constriction and heart function (Beutner et al., 2005; Eisner et al., 2017; Lascano et al., 2017; Mayourian et al., 2018). Carbonylation is the most prevalent non-enzymatic post-translational modification (PTM) observed in a murine model of type 1 diabetes. PTMs by AGEs and ROS cause the variation in RyR2 activity found in preclinical models of diabetes (Shao et al., 2012; Tian and Zhen, 2019). Several studies published over the past 10 years suggest that increased RyR2 expression may contribute to the pathogenesis of cardiac dysfunction. Impact of RyR2 potentiation on myocardial function (Bockus and Humphries, 2015; Lascano et al., 2017; Tian and Zhen, 2019). RCS and AGEs are produced due to hyperglycemic alterations of the regular metabolic biochemical pathways, accumulating on specific basic residues. Through RyR channels, mutations of these residues affect the physicochemical properties of carbonyl and oxidative stress, thereby influencing whether cytoplasmic Ca^2+^ responsiveness is increased or decreased. Cardiomyocyte, fibroblast, and immune system dysfunction contribute to the pathogenesis of DCM and HF. Inappropriate immunological responses and poor cardiac E-C coupling are the results of leaky RyR2 channels, which alter the ECM output and aberrant fibroblastic features.

TGF has been shown to affect the function of cardiomyocytes directly and is a critical mediator in converting cardiac fibroblasts into myofibroblasts. There is also evidence that IL6 is involved in the communication mechanisms (Cartledge et al., 2015; Pellman et al., 2016; Hall et al., 2021). Increased oxidative stress, brought on by the production of ROS, is associated with diabetic problems. These substances may have the capacity to interact with reactive cysteines, leading to RyR2 malfunction. TNF-α and IL-1β promote Ca^2+^ efflux from the SR that contributes to suppressed Ca^2+^ transients and causes arrhythmia in the ability of rat ventricular myocytes to contract (Duncan et al., 2010; Szekely and Arbel, 2018). Immune-related chemicals, including TNF-α and IL-1β, replicate harmful effects on activated SR. RyR2 clusters engaged in the calcium transients might resist induced CICR because of the Ca^2+^ release from the SR.

Cardiomyocytes with low levels of miR-22 exhibited aberrant SR Ca^2+^ handling, characterised by reduced PLN phosphorylation, decreased SERCA2a transport activity, and lower SERCA2a levels. Purine-rich element-binding protein B (PURB) and Sirtuin 1 are two examples of miR-22 target genes identified (Gurha et al., 2012; Gurha et al., 2013). SRF, a transcriptional regulator of the *SERCA2a* gene and hypertrophic genes, is repressed by PURB. According to Gurha et al. (2012), Wahlquist et al. (2014), during HF, miR-25 is upregulated, followed by a decrease in SERCA2a expression.

Overexpression of miR-25 in mouse cardiac muscle cells decreased the cardiac capacity to contract, while miR-25 suppression through the injection of anti-miR-25 oligonucleotides (antagomiR) restored heart health and extended survival in HF models. By delivering Adeno-associated virus (AAV) vectors encoding an anti-miR-25 tough decoy (TuD) *in vivo*, subsequent investigations have shown that miR-25 may be effectively suppressed over an extended period (Jeong et al., 2018). In contrast, reducing miR-25 levels with anti-miR-25 oligonucleotide injection increased cardiac activity and survival rates in mice following HFD. After establishing that increased levels of cardiac miR-25 through AAV9 resulted in reduced expression of SERCA2a and impaired contractile function, the researchers demonstrated that administering anti-miR-25 oligonucleotide therapeutically could reverse pre-existing HF in mice with thoracic aortic constriction (TAC), even in the presence of persistent pressure overload (Bush and Van Rooij, 2014). The inhibitory action of miR-25 on SERCA2a and potentially IP3R1 contributes to its protective effect without affecting other important calcium-handling proteins such as phospholamban or the NCX (Wahlquist et al., 2014).

miR-146a is expressed in endothelial cells that regulates the function of the SERCA2a pump (Feng et al., 2017). Immune response has been linked to miR-146. The expression of SUMO-1 (small ubiquitin-related modifier) is found to regulate the activation of the unfolded protein response and endoplasmic reticulum homeostasis, upregulating the SERCA2a pump, which was inversely linked with miR-146a transcript levels, which rose in both animals and HF patients. In *in vitro* tests, miR-146a overexpressed cardiomyocytes’ Ca^2+^ transient decay was extended because sumo1 expression was lower. An effective biomarker for assessing the effectiveness of anti-HF drugs is miR-146a (Moreira-Costa et al., 2021).

## NF-κB

NF-κB belongs to the family of Rel, the five members of the ubiquitous family of inducible dimeric homodimer or heterodimer transcription factors. These proteins contain a 300-amino-acid Rel homology region that is highly conserved and comprises two immunoglobulin-like domains (Giridharan and Srinivasan, 2018). NF-κB was first discovered to be a nuclear factor linked to the upregulation of the B-lymphocytes immunoglobulin-light chain gene. Nevertheless, it is evident that a large number of NF-κB:IκB complexes may be present within the cells, providing a vast array of options and permitting specific NF-κB system controls. As a result, various activated NF-κB dimers bind to distinct consensus B sites in the heart with varying affinities (Sasaki and Iwai, 2015). The B sites can be unequal, followed by subtle cell- and gene-specific modulations in gene expression. The cAMP Response Element Binding Protein (CREB) signal transducers and transcription adaptors are two important coactivators that work together to increase gene transcription, not NF-κB dimers alone (Li et al., 2008). NF-κB DNA binding is inhibited (and I-B levels are increased) by the physical interaction of NF-κB with the glucocorticoid receptor, another transcription factor (Riedlinger et al., 2019). Another transcription factor that can prevent the nuclear translocation of NF-κB is the PPAR (Smeets et al., 2008). NF-κB regulates numerous biological processes, including immunological responses, stress responses, surface receptor signalling, cell viability, growth factor regulation, cell division, and genes associated with the ubiquitin-proteasome system (Li et al., 2008).

MAP-kinases/PPAR activation is a possible mechanism in the heart pro-hypertrophic NF-κB-dependent pathway. In p50 null mice, the MAP-kinase-mediated CH caused by AT II was reduced. MAP-kinase and NF-κB blockage may also improve the shift in VH from glucose metabolism to FA breakdown (or PPAR activation). In agreement, we have found that incubation with a PPAR agonist inhibits hypertrophy in glucose-treated cardiomyocytes. Notably, mice lacking NF-κB interacting Protein-1, an NF-κB repressor, exhibited a more severe progression of dilated cardiomyopathy but not VH (Herron et al., 2005). Myocardial I/R damage can elevate p-TLR4 and p-NF-κB expression levels (Pan et al., 2018). TLR4 is abundantly present in cardiac cells and microvascular endothelial cells, and it can enhance the production of interferon-β and trigger the activation of NF-κB via the MyD88-dependent pathway. In circumstances such as ischemia and hypoxia, the level of phosphorylated TLR4 increases, leading to the activation of TLR4. This activation further enhances the phosphorylation of NF-κB, triggering the TLR4/NF-κB pathway and increasing the production of inflammatory components (Pan et al., 2018).

Although miR-21 is expressed in various tissues, it is most abundant in cardiac tissue. miR-21 operates on TLR4 to mediate apoptosis in numerous physiological and pathological processes. TLR4 is one of the target genes of miR-21. Overexpressed miR-21 dramatically reduced myocardial apoptosis and apoptotic protein expression, effectively downregulated the release of inflammatory factors, and successfully enhanced cardiac function (Pan et al., 2018). Myocardial cell injury resulting from myocardial ischemic injury is effectively reduced by the upregulation of miR-21, which inhibits the TLR4/NF-κB pathway and decreases the expression levels of p-TLR4 and p-NF-κB, as previously discussed.

In the cardiac tissues of diabetic murine models and HG-induced cardiomyocytes, miR-92a-2-5p was found to be downregulated. In diabetic rats, miR-92a-2-5p treatment enhanced heart remodelling and function (Yu et al., 2022). Upregulation of miR-92a-2-5p reduced ROS accumulation in cardiac cells, adversely affecting MKNK2, one of the two serine/threonine kinases that are substrates in the MAPK pathway. Additionally, it is essential to mention that MAPK is recognised as an inducer of inflammation in DCM, as it amplifies NF-κB expression, hence intensifying DCM by enhancing inflammation and the generation of pro-inflammatory cytokines (Rezaee et al., 2024). These findings imply an intense, synchronised myocardium stress programme involving translational activation in the mitochondria and inhibition in the cytoplasm. According to research, the p38-MAPK pathway can be activated by the MKNK2a isoform to cause apoptosis but not by the MKNK2b isoform (Maimon et al., 2014). The 3′UTR of the human MKNK2a isoform contained two putative miR-92a-2-5p binding sites, while the MKNK2b isoform lacked any such sites, according to bioinformatics screening. These findings suggest that miR-92a-2-5p may regulate MKNK2a function in humans and could be an essential regulator of the MKNK2a-to-MKNK2b expression ratio. However, there are also many miRNAs involved in the molecular pathogenesis of DCM, as mentioned in Table 2.

**TABLE 2:** List of chosen miR-expression in various target sites of DCM models.

Sl. No.	miRNA	Expression in disease condition	Research model	Targeting genes/signalling pathway	Pathophysiological condition	References
1	miR-1/206	Upregulated	STZ-diabetic rat and mouse heart	Pim-1, Hsp60	Cardiac apoptosis	Shan et al. (2010)
2	miR-9	Downregulated	Human diabetic heart IHVC	ELAVL1	Cardiac structural damage	Jeyabal et al. (2016)
3	miR-133a	Downregulated	STZ-diabetic mouse heart	SGK1, IGF-1R, TGF-β1	Cardiac hypertrophy (CH) and fibrosis	Feng et al. (2010), Chen et al. (2014)
4	miR-29	Upregulated	ZDF rat heart	MCL-1	Cardiac structural damage	Arnold et al. (2014)
5	miR-34a	Upregulated	H9c2 cells	BCL-2, SIRT-1	Cardiac apoptosis	Zhao et al. (2013), Fomison-Nurse et al. (2018)
6	miR-451	Upregulated	HFD-induced mouse heart	CAB-39	CH	Kuwabara et al. (2015)
7	miR-483-3p	Upregulated	H9c2 cells, STZ-mouse heart	IGF-1, BCL-2	Cardiac apoptosis	Qiao et al. (2016)
8	miR-373	Downregulated	STZ- diabetic mouse heart	MEF2c	CH and oxidative stress	Shen et al. (2011), Yildirim et al. (2013)
9	miR-208a	Upregulated	STZ-diabetic mouse	GATA4, Thrap-1	CH	Moore et al. (2014)
10	miR-144	Downregulated	STZ-mouse, HG-treated cardiomyocyte	Nrf2	Cardiac apoptosis and oxidative stress	Yu et al. (2015)
11	miR-195	Upregulated	STZ-C57BL/6 mice	BCL-2 and Sirt1	Cardiomyocytes apoptosis	Zheng et al. (2015)
12	miR-212	Upregulated	STZ-C57/B6 mice	calcineurin/NFAT signalling	Hypertrophy and autophagic response	Ucar et al. (2012), Costantino et al. (2016)
13	miR-221	Upregulated	STZ-C57/B6 mice	p27/mTOR	Hypertrophy and autophagic response	Costantino et al. (2016)
14	miR-320	Upregulated	HFD-miR-320–overexpressing tg mice	CD36	Cardiomyocytes apoptosis	Li et al. (2019)
15	miR-378	Downregulated	STZ-diabetic mouse, Mouse osteoblastic cell line MC3T3-E1	CASP3, PI3K/Akt signalling pathway	Hypertrophy and oxidative stress	You et al. (2014)
16	miR-125b	Downregulated	C57BL/6 mice	Bcl-2, BAK-1	Cardiomyocytes apoptosis	Zhang et al. (2021a)
17	miR-150	Downregulated	STZ-diabetic rat	p300	Hypertrophy	Duan et al. (2013)
18	miR-199a	Downregulated	tg mouse	mTOR signalling and Atg5, Atg12, Beclin1 and LC3B genes	Cardiac autophagy, hypertrophy	Li et al. (2017b)
19	miR-30c	Downregulated	H9c2 and HEK293T cell line STZ-diabetic mouse	PGC-1β	Cardiomyocyte apoptosis and cardiac dysfunction	Yin et al. (2019)
20	miR-499	Upregulated	STZ-diabetic mouse	Gadd45alpha	Baroreflex dysfunction and myocardial damage	Wang et al. (2012)
21	miR-210	Downregulated	Humane and KO mouse	protein tyrosine phosphatase 1B (PTP1B)	Endothelial dysfunction	Zhou et al. (2022)
22	miR-27a-3p	Upregulated	H9c2 cell line	NOVA1	CH	Li et al. (2022a)
23	miR-30a	Upregulated	STZ-diabetic mouse	Bcl-2, Sirt-1	Cardiomyocyte apoptosis and fibrosis	Bernardo et al. (2022)
24	miR-214-3p	Downregulated	Human	death-associated protein kinase-2	Cardiomyocyte apoptosis	Qi et al. (2020)
25	miR-146a	Downregulated	STZ-diabetic mouse	p300	Cardiomyocyte apoptosis and fibrosis	Feng et al. (2017)
26	miR-340-5p	Upregulated	STZ-diabetic mouse	Mlc-1	cardiac oxidative stress, mitochondrial function, and apoptosis	Zhu et al. (2022)
27	miR-200c	Upregulated	HFD and low dose STZ induced mouse	DUSP-1	CH	Singh et al. (2017)
28	miR-181b	Downregulated	HFD-induced mouse heart	TGF-β/pSmadD2/3 pathways	Cardiac fibrosis	Uribe et al. (2017)
29	miR-223-3p	Upregulated	H9c2 rat cardiomyoblast cell line	SPI1	Cardiomyocyte pyroptosis	Zhao et al. (2022)
30	miR-155	Upregulated	STZ-diabetic mouse	Nrf2	Cardiomyocyte apoptosis	Jankauskas et al. (2021)

## miRNAs in cardiac immunity of DCM

**FIGURE 3:**
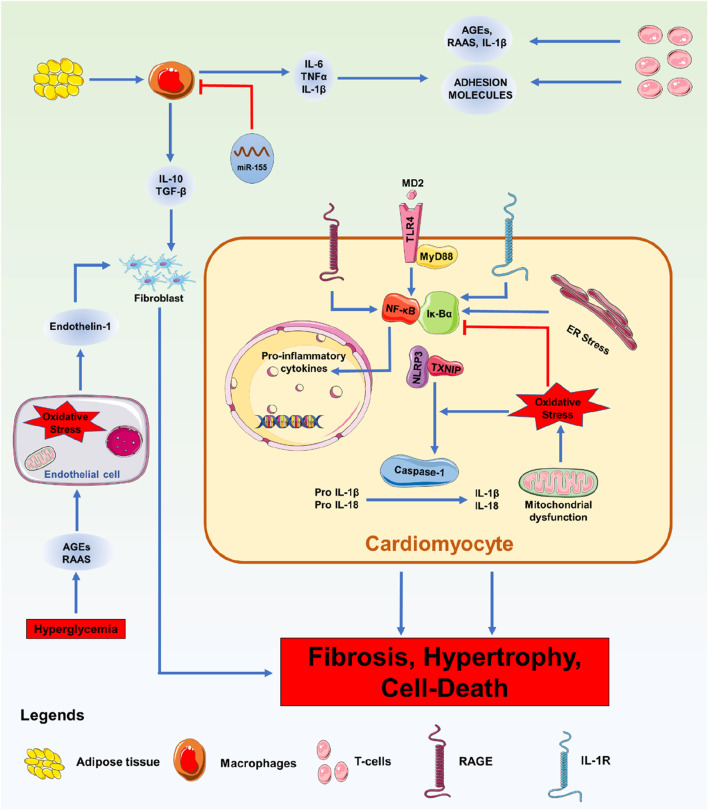
Immunological Phenomena with miRNA: Overview of the signalling processes involved in DM-related cardiac inflammation. Enhanced leukocyte migration in the myocardium is a feature of DM. Pathological stressors such as high blood sugar, high cholesterol, increased RAAS, and AGEs cause leukocytes to secrete pro-inflammatory chemicals, adhesion molecules, and DAMPs. Additionally, these catalysts cause endothelial dysfunction brought on by ROS, which aids in heart remodelling. Pro-inflammatory cytokines are secreted, and when they bind to receptors, including the TLR-4-MyD88 complex, RAGE, and IL-1R, they initiate intracellular signalling cascades. Inflammatory cytokines and the NLRP3 inflammasome are transcriptionally upregulated due to the activation of NF-κB by these pathways. Inflammasome assembly follows the activation of NF-κB and oxidative stress, which trigger the maturation of IL-1β and IL-18 and the induction of pyroptosis.

Macrophages play a crucial role in regulating immune system functions and are essential for controlling inflammation. Inflammation can be treated by activating M2 cells, but M1 cells are known to generate proinflammatory cytokines when activated (Martinez et al., 2008). In situations where miR-155 is known to be increased, the miR-155 inhibitor antagomiR-155 has been shown to decrease the flow of pro-inflammatory cytokines into the heart, limit myocardial damage, and improve cardiac function (Corsten et al., 2012). The pro-inflammatory type of DCM animals developed more inflammation as a result of high M1 macrophage infiltration induced by oestrogen deprivation (Figure 3). Instead of causing DCM to worsen due to an estrogen shortage, gold nanoparticle-conjugated antagomiR-155 treatment promoted the infiltration of M2-type macrophages and enhanced cardiac shape and function. It has been proposed that miR-155 suppression therapy could be a valuable strategy to enhance heart function in DCM (Jia et al., 2017). In DCM macrophages, efferocytosis reduces the phagocytic clearance of apoptotic cells. miR-126 was discovered to alter the phagocytosis of apoptotic myocytes by macrophages (Suresh Babu et al., 2016). In macrophages under hyperglycemic circumstances, miR-126 expression was downregulated, whereas the A Disintegrin And Metalloprotease (ADAM)*-*9 gene expression was upregulated. Membrane-anchored enzymes, known as ADAMs, are involved in several cellular activities, including cell migration, the release of cytokines and growth factors, and the inflammatory response. Similarly, miR-126 overexpression reduces the malfunction of efferocytosis. Pharmaceutical treatment that enhances the expression of miR-126 in this pathway can improve cardiac muscle function after damage and under inflammatory conditions associated with DM (Shen et al., 2020). T2DM patients have downregulated GLUT4 expression and have upregulated miR-223.

Interestingly, mice with pathological cardiac events like MI and cardiac ischemia have greater levels of miR-223 expression (Van Rooij et al., 2008). The miR-223 has been found to have a myeloid-specific expression pattern in the immune system, and this expression depends on the granulopoiesis regulator CCAAT/enhancer-binding protein (C/EBP) being active (Fazi et al., 2007). Similarly, it also showed that animals lacking miR-223 produced, differentiated, and activated more granulocytes, which triggered inflammation and accelerated tissue damage. Consequently, miR-223 controls the production of granulocytes and the inflammatory response (van de Vrie et al., 2011).

## Theranostic values of miRNAs in management of DCM

miRNA has potential and significant roles in epigenetic modulation and the regulation of various critical protein factors, so it may also serve as a crucial diagnostic biomarker and therapeutic agent, combining theranostic value in the management of DCM.

## Diagnostic role

Blanco-Domínguez and colleagues (Blanco-Domínguez et al., 2021) discovered a novel miRNA, offering a new perspective for the development of fast diagnostic kits in clinical settings. Moreover, current miRNA microarray data reveal minimal overlap in the identification of miRNAs linked to various types of DM (León et al., 2016). Consequently, exosomal miRNAs have garnered interest as a potential biomarker for the diagnosis of DCM. Furthermore, advancements in technology, such as two-dimensional bismuth alkene nanosheet probes, facilitate the detection of miRNA for early detection. Myocardial miRNAs, including miR-1, miR-208, and miR-499, are highly expressed in circulating exosomes, making their alterations a strong indicator of myocardial damage (Cheng et al., 2019). Furthermore, circulating concentrations of miR-19b-3p and miR-181b-5p exhibit a favourable correlation with the myocardial condition during the progression of DCM. Costantino et al. demonstrated that 316 of 1,008 miRNAs are dysregulated in the hearts of diabetic mice, with several miRNAs being found in human plasma exosomes (Huang et al., 2013). The expressions of miR-126 and miR-320 were elevated in patients with DCM. miR-320 can inhibit angiogenesis and enhance heart function (Wang et al., 2014). The findings suggest that exosomal miRNAs have potential diagnostic significance in CVD.

The statistical study of many exosomal miRNAs might enhance their diagnostic credibility. Consequently, the integration of diverse exosomal miRNA data will enhance diagnostic efficacy.

## Therapeutic value

Exosomal miRNA has superior affinity and targeting compared to traditional miRNA carriers. Furthermore, several proteins, including CD47, present on the surface of exosomes, inhibit their uptake by monocytes or macrophages; hence, the miRNAs contained within exosomes have an extended half-life *in vivo*. Wang et al. demonstrated that modified exosomes can selectively target ischemic myocardium and improve treatment outcomes in myocardial infarction (Wang et al., 2018). Exosomal miR-130a-3p, originating from the liver, enhances sensitivity to insulin and the breakdown of glucose by inhibiting the expression of the PHLPP2 gene in adipocytes affected by metabolic diseases (Wu et al., 2020). Exosomal miRNAs generated from adipose tissue, such as miR-99b, can enhance glucose tolerance and rectify lipid metabolic disorders [62]. The cardioprotective actions of exosomes are largely ascribed to miRNAs, such as miR-15b, miR-17, miR-20a, miR-103, miR-199a, miR-210, and miR-292 (Gray et al., 2015).

Additionally, nanomedicine is offering new hope in the treatment of DCM, a condition marked by progressive heart muscle damage, including fibrosis, inflammation, and cell death. A key contributor to DCM is the abnormal expression of miRNAs. Among these, miR-133a, miR-34a, miR-155, and miR-146a have been identified as playing central roles in driving disease progression. Despite their therapeutic potential, the use of miRNAs in treatment has been challenging due to their instability, rapid degradation in the bloodstream, and lack of targeted delivery. This is where nanomedicine makes a significant difference. By utilising specialised carriers, including exosomes and nanoparticles, researchers can protect miRNAs from degradation and direct them to specific tissues, thereby enhancing both stability and therapeutic efficacy. Exosomal miRNAs can rectify aberrant electrical activity. Cardiac ECM-derived EVs containing miR-199a-3p can augment myocardial electrical signalling activity by promoting the acetylation of GATA-binding protein 4 (An et al., 2017). Furthermore, exosomes contribute to the prevention of DCM by lifestyle modifications, including aerobic activity, weight management, and smoking cessation. Despite being derived from animal models, exosomal miRNAs demonstrate potential efficacy in the treatment of DCM. A recent study revealed that in diabetic models, small extracellular vesicles (sEVs) from heart cells exhibited a decline in miR-194-3p levels, which contributed to cardiac fibrosis. Restoring this miRNA through exosome-based delivery helped reduce fibrosis and improve heart function in diabetic mice (Li et al., 2024).

In contrast, gold nanoparticles (AuNPs) have also been used to deliver miRNA antagonists in DCM. In one example, gold nanoparticles were conjugated with antagomiR-155 and administered to diabetic mice. The treatment significantly reduced heart inflammation and improved overall cardiac function. Similarly, another promising platform involves polymer-based nanoparticles, such as poly (lactic-co-glycolic acid) (PLGA). These carriers can provide a controlled, sustained release of miRNA therapeutics. PLGA nanoparticles loaded with antagomiR-92 have been shown to promote new blood vessel formation (angiogenesis) and enhance cardiac function in diabetic mice recovering from heart attacks (Macvanin and Isenovic, 2023). Further research is required before its clinical application can be considered. However, in the present day, miRNAs undergo various clinical trials to investigate better therapeutic strategies for the management of diabetes and cardiovascular diseases.

## Role of “calcium-signalling” as a theranostic approach for the DCM

Ca^2+^ signalling dysfunction is widely acknowledged as a critical factor in the initiation and advancement of DCM. Chronic hyperglycaemia and insulin resistance in diabetes impair calcium cycling in cardiomyocytes by reducing SERCA2a activity, increasing RyR2 leakiness, and promoting mitochondrial calcium overload, ultimately leading to diastolic dysfunction and myocardial fibrosis (Sun et al., 2025). Theranostically, the fluorescent Ca^2+^ sensitive dyes, Fura Red and Fluo-4 are commonly used for visual measurement of Ca^2+^ homeostasis *in vitro*. The fluorescence behaviour of these dyes is greatly dependent on temperature, pH, and ionic strength. Moreover, some small synthetic dyes, such as Fura-2 and Fluo-3, as well as several groups of genetically encoded calcium indicators (GECIs) based on GFP-like fluorescent proteins, are also considered in Ca^2+^ imaging (Simonyan et al., 2024). An experiment measured transient [Ca^2+^] in isolated myocardial cells from diabetic rats using Fura-2 dye (Sheikh et al., 2012). However, genetically encoded Ca^2+^ markers, such as GCaMP6, have made it possible to track the flow of Ca^2+^ inside cells in real time in preclinical models, especially in human iPSC-derived cardiomyocytes under hyperglycemic stress (Garcia et al., 2017; Simonyan et al., 2024). Recently, a study showed that GCaMP6s-BrUS-145, which is genetically engineered by GCaMP6 backbone and the fluorescent protein BrUSLEE, can be used for static measures, especially when using fluorescence lifetime imaging microscopy (FLIM) to find the absolute intercellular Ca^2+^concentration values (Simonyan et al., 2024).

Calcium channel blockers (CCBs) may provide further advantages to diabetic patients by reducing cardiac complications and rectifying intracellular calcium abnormalities that could play a role in the pathogenesis of DCM. Non-dihydropyridine CCB verapamil has been seen as a potential new way to treat diabetes and associated cardiac problems. Antidiabetic efficacy of R-form verapamil enantiomer (R-Vera) and S-form verapamil enantiomer (S-Vera) was measured on STZ-induced C57BL/6J mice models (Chen et al., 2021). R-Vera appears to be an effective alternative in diabetes treatment by lowering β-cell death and blocking calcium channels. It also prevents long-term β-cell impairment, which is partly caused by chronic increased intracellular Ca^2+^ levels. However, another study on STZ-induced SD rats and found istaroxime to be a promising therapeutic approach by stimulating SERCA2a. This agent accelerates Ca^2+^ uptake into the sarcoplasmic reticulum (SR) through the SR Ca^2+^ pump and also inhibits Na^+^/K^+^ ATPase, which integrates to improve intracellular Ca^2+^ handling and diastolic dysfunction in a DCM model (Torre et al., 2022).

## Role of imaging techniques in DCM

Theranostics integrates diagnosis and therapy into a unified approach for disease management. In the context of DCM, the imaging technologies form the diagnostic arm of this paradigm, enabling the detection of structural, functional, metabolic, and even molecular changes in cardiac tissues, often preceding symptomatic manifestations.

## Echocardiography

Echocardiography remains the primary diagnostic tool in clinical settings for DCM due to its accessibility and non-invasive nature. It provides insight into ventricular wall thickness, ejection fraction, diastolic dysfunction, and myocardial strain. Advanced echocardiographic modalities, such as speckle-tracking imaging, can detect subclinical myocardial dysfunction even in asymptomatic diabetic patients, making them a valuable tool for longitudinal monitoring (Urlic et al., 2023).

## Cardiac magnetic resonance imaging (CMR)

CMR is considered the gold standard for non-invasive quantification of cardiac morphology, function, and fibrosis. Techniques such as T1 and T2 mapping, as well as late gadolinium enhancement (LGE), enable the detection of diffuse interstitial fibrosis, a hallmark of DCM, and provide prognostic value. CMR can also track changes in myocardial tissue composition following miRNA-based or other therapeutic interventions, thereby serving as a feedback tool for therapeutic efficacy (Liu X. et al., 2022; Marey et al., 2025).

## Positron emission tomography (PET)

PET imaging using radiotracers such as [^18F] FDG (for glucose metabolism) and [^11C] palmitate or [^13N] ammonia (for fatty acid metabolism and perfusion) can characterise the metabolic shifts in diabetic myocardium. Since metabolic dysregulation is central to DCM, PET imaging aids in both the early detection and evaluation of response to metabolic therapies, including those targeting miRNA pathways (Hsieh et al., 2024).

## Molecular imaging and miRNA theranostics

Emerging molecular imaging platforms are being developed to visualise miRNA activity *in vivo* using labelled oligonucleotide probes or miRNA-responsive nanoprobes. These technologies enable the spatial and temporal mapping of miRNA expression, allowing for the real-time assessment of miRNA dysregulation in cardiac tissues. Integration with therapeutic nanocarriers creates a feedback loop, as the same platform can deliver therapy and monitor its effects, aligning perfectly with the theranostic concept (Mathur and Rani, 2023).

The future of imaging in DCM theranostics lies in hybrid modalities, such as PET-MRI, and artificial intelligence (AI)-aided image interpretation, which can correlate imaging phenotypes with molecular miRNA signatures. The integration of imaging biomarkers with circulating exosomal miRNAs holds promise for non-invasive, personalised disease management.

## Other emerging diagnostic and therapeutic strategies for DCM

Other emerging therapeutic and diagnostic strategies have focused on molecular mechanisms, including messenger RNA (mRNA), CRISPR-based gene editing, and small-molecule drugs. Each of these approaches has distinct mechanisms, benefits, and limitations in its application to DCM.

## mRNA-based therapy

mRNA therapy involves the delivery of synthetic mRNA into cells to produce therapeutic proteins. This technology provides a transient and controllable method for enhancing protein expression (Qin et al., 2022). In DCM, mRNA could be used to express cardioprotective proteins such as VEGF (for angiogenesis), SOD2 (for reducing oxidative stress), or SERCA2a (for improving calcium handling) (Chen and Guo, 2025). Unlike miRNAs that modulate endogenous pathways, mRNA introduces new functional proteins directly. While mRNA-based diagnostics are less common, transcriptomic profiling of cardiac or blood samples can reveal mRNA signatures of DCM progression. However, mRNA therapy faces challenges in stability, immune activation, and delivery to cardiomyocytes, although lipid nanoparticle (LNP) systems have improved delivery efficiency (Parikh et al., 2022).

## CRISPR-based gene editing

CRISPR-Cas9 enables the creation of precise, permanent genome modifications by targeting specific DNA sequences (Aljabali et al., 2024). In the context of DCM, CRISPR could potentially correct pathogenic mutations (e.g., in the insulin signalling pathway, mitochondrial regulators, or fibrosis-related genes), offering a curative approach (Kambis and Mishra, 2023). For instance, targeting the TGF-β signalling cascade, as well as oxidative stress and AGEs (advanced glycation end products)-associated genes, could attenuate fibrosis and inflammation. Unlike miRNA or mRNA, CRISPR modifies the genome itself, which may lead to long-term effects with a single treatment (Chen et al., 2024). However, this permanence raises safety concerns, including off-target edits, immunogenicity, and ethical considerations. Although CRISPR is still largely preclinical for cardiac applications and lacks standardised delivery systems to the heart (Aljabali et al., 2024).

## Small-molecule drugs

The renin-angiotensin-aldosterone system (RAAS) plays a central role in the development of DCM by promoting myocardial fibrosis, hypertrophy, oxidative stress, and inflammation. Chronic hyperglycemia upregulates RAAS activity, particularly angiotensin II and aldosterone, which activate pro-fibrotic (e.g., TGF-β/Smad), pro-hypertrophic (MAPK/ERK), and pro-inflammatory (NF-κB) signalling pathways in cardiomyocytes. Recent works targeting angiotensinogen messenger RNA (mRNA) synthesis in human heart tissue confirmed that angiotensinogen is synthesised locally in the human heart, highlighting the heart’s intrinsic RAAS activity. In fact, RAS blockade resulted in upregulated plasma Ang-(1–7) and ACE2 mRNA in heart tissue, suggesting a shift toward cardioprotective signalling. In the context of RAAS and DCM, specific miRNAs have also been found to be dysregulated and associated with disease progression. For example, miRNA-21, miRNA-29, miRNA-133, and miRNA-155 are associated with the regulation of RAAS components and pathways involved in cardiac remodelling, fibrosis, inflammation, and hypertrophy. These miRNAs can directly modulate the expression of genes related to the RAAS pathway, including ACE, the AT1 receptor, and other critical signalling molecules (Batista et al., 2023). Pharmacological agents, such as ACE inhibitors (ramipril) and ARBs (valsartan), help alleviate DCM by inhibiting angiotensin II signalling, thereby suppressing the TGF-β and MAPK pathways and reducing myocardial fibrosis and hypertrophy. SGLT2 inhibitors (such as dapagliflozin), although primarily used for glycemic control, indirectly modulate RAAS activity and reduce oxidative stress and inflammation through NLRP3 inflammasome inhibition and lowered ROS production (Batista et al., 2023; Vlachakis et al., 2024).

Oxidative stress in diabetic hearts, due to elevated reactive oxygen species (ROS), disrupts cellular signalling, leading to protein, lipid, and DNA damage, as well as endoplasmic reticulum stress and oxidative changes in microRNAs, ultimately causing cardiomyocyte dysfunction or death via necrosis or apoptosis. ROS activate stress-sensitive pathways such as JNKs, ERK1/2, p38 MAPK, NF-κB, and PKC, contributing to myocardial injury. High ROS levels impair calcium handling, promote hypertrophy, and increase interstitial and perivascular fibrosis. Notably, apoptosis is significantly elevated in the myocardium of diabetic patients, underscoring oxidative stress as a central driver of DCM progression (De Geest and Mishra, 2022). However, these small synthetic molecules have conventionally dominated pharmacological interventions due to their ease of synthesis, oral bioavailability, and cost-effectiveness. Still, their lack of specificity compared to miRNA or gene-editing approaches often results in undesired actions and suboptimal targeting of the molecular root causes of DCM. Additionally, they do not address the genetic or epigenetic alterations that underlie disease progression.

## Influence of demographic and physiological factors on miRNA for clinical biomarker development

miRNA expression patterns are known to vary significantly across individuals due to inherent biological differences. In the context of using miRNAs as clinical biomarkers for DCM, the influence of demographic and physiological factors—such as age, gender, and comorbidities—remains an underexplored yet critical aspect.

Ageing significantly influences the molecular landscape of the cardiovascular system, including the expression patterns of miRNAs that regulate critical biological processes such as cardiac remodelling, inflammation, and oxidative stress, each of which plays a central role in the pathogenesis of DCM. As individuals age, the expression levels of several key miRNAs undergo notable shifts, potentially altering the balance of pro-fibrotic, pro-inflammatory, and apoptotic signalling within cardiac tissue. These age-related miRNA-derived changes can either exacerbate or attenuate the progression of DCM, depending on the specific regulatory pathways involved. Therefore, failing to consider age as a variable when analysing miRNA profiles in diabetic patients could lead to misleading interpretations. For example, a miRNA upregulated due to ageing might be incorrectly attributed to diabetic pathology, or *vice versa*; a miRNA with diagnostic or prognostic potential might be overlooked because its expression appears variable across age groups. Thus, integrating age-stratified analysis is essential not only to improve the accuracy of miRNA-based biomarkers but also to refine therapeutic strategies tailored to different age populations. Without such consideration, there is a substantial risk of developing generalised models that lack clinical sensitivity and specificity, ultimately hindering the translational success of miRNA-targeted approaches for DCM (Florio et al., 2020).

Gender also plays a pivotal role in miRNA dynamics, as hormonal differences can significantly modulate the expression, processing, and stability of specific miRNAs implicated in cardiovascular function. Estrogen, for example, has been shown to influence the biogenesis of various miRNAs through estrogen receptor-mediated signalling pathways, affecting the transcription of miRNA genes as well as the activity of enzymes such as Drosha and Dicer involved in miRNA maturation. For instance, certain cardioprotective miRNAs may be upregulated in premenopausal women due to higher estrogen levels, offering some degree of natural protection against DCM. In contrast, the absence of this hormonal influence in males or postmenopausal females could contribute to more severe cardiac remodelling and dysfunction. Likewise, testosterone can regulate cardiac gene expression indirectly by altering miRNA profiles associated with myocardial hypertrophy and fibrosis. These hormonal effects translate into gender-specific patterns of miRNA expression in both healthy and diseased hearts. Furthermore, miRNAs such as miR-1, miR-133a and b, and miR-208, which are closely associated with cardiac contractility and hypertrophy, have demonstrated sex-specific expression patterns in animal models of cardiac disease (Boštjančič et al., 2010). Ignoring these gender-based differences when analysing miRNA data in DCM may lead to inconsistent findings or the masking of potential biomarkers that are only significant within one sex. This lack of stratification could ultimately compromise the diagnostic accuracy and therapeutic potential of miRNA-based tools. Therefore, it is imperative to include gender as a key variable in future biomarker discovery studies, ensuring the development of personalised and effective diagnostic and treatment strategies for DCM (Toedebusch et al., 2018).

Furthermore, comorbid conditions commonly associated with diabetes, such as hypertension, obesity, and dyslipidemia, may confound miRNA expression profiles. These overlapping pathologies can lead to shared or competing miRNA regulatory pathways, thereby masking DCM-specific signatures. For example, suppression of miR-21 and miR-155 have shown promising effects in diabetic heart failure. Ignoring such variables limits the clinical utility of miRNAs as precise, disease-targeted biomarkers for DCM (Macvanin M. T. et al., 2023).

To develop robust miRNA-based diagnostics or therapeutics for DCM, future research must rigorously account for these demographic and physiological variables. Stratified study designs, larger population-based cohorts, and integrative analyses incorporating age, sex, and comorbidity profiles will be crucial for validating miRNA candidates with high sensitivity and specificity. Only through such comprehensive evaluation can miRNAs be reliably translated into clinical practice for managing DCM.

## Recent advances in clinical trials and translational studies of miRNA therapeutics in DCM and CVD

Recent advancements in clinical trials investigating miRNAs in DCM have emphasised their potential as diagnostic indicators and therapeutic targets. Recent research has placed greater emphasis on specific miRNAs involved in the development of DCM. These trials have shown encouraging outcomes, indicating a connection between modified levels of expression of these miRNAs and CVD in individuals with diabetes. In addition, new treatment approaches using miRNA-based medicines, including antagomirs and mimics, are now being explored to regulate the production of these miRNAs. CDR132L, an antisense oligonucleotide targeting miR-132, has shown promising results in phase I clinical trials for patients with heart failure. In a randomised, placebo-controlled trial (NCT04045405), patients receiving CDR132L demonstrated improved cardiac function, reduced NT-proBNP levels, and a favourable safety profile. Since miR-132 is involved in cardiac hypertrophy and remodelling, this trial offers valuable translational insights for miRNA-based modulation in diabetic cardiac pathology (Täubel et al., 2021).

The antimiR-92a agent MRG-110 has advanced to early-phase clinical trials (NCT03603431). In healthy volunteers, MRG-110 successfully suppressed circulating miR-92a levels and improved markers of vascular repair and endothelial regeneration. Since impaired vascular function plays a crucial role in DCM progression, these findings support the therapeutic relevance of endothelial-targeted miRNA therapies (Huang et al., 2020). Circulating levels of miR-126 and miR-210 are associated with endothelial dysfunction in patients with T2DM and may predict subclinical cardiac dysfunction, potentially allowing for earlier intervention (Amr et al., 2018). Moreover, recent investigations have explored exosome-derived miRNAs as highly stable biomarkers for diabetic cardiovascular complications. In a prospective cohort study, elevated circulating exosomal miR-423-5p and miR-320a were associated with early myocardial remodelling in diabetic patients with preserved ejection fraction, suggesting their potential role in guiding clinical decision-making (Delshad et al., 2025).

A large prospective clinical trial (NCT04889053) is recruiting 1,400 diabetic patients to evaluate circulating miRNA signatures associated with vascular calcification and coronary artery disease. Initial findings suggest that miR-32-5p and miR-33 levels correlate with progression of coronary calcification, supporting the concept of miRNA-guided cardiovascular risk stratification in diabetic patients (https://clinicaltrials.gov/ct2/show/NCT04889053).

A summary of the clinical studies involving miRNAs in CVD linked to diabetes has been described in Table 3.

**TABLE 3:** Clinical trials data on diabetes and related cardiac disorders associated with miRNAs.

Clinical trial No	No of partici-pants	Condition	Study type	Status	Comments	References
NCT03792607	35	T2DM, CVD	Observational/Prospective	Unknown	Diagnosis of CVD in T2DM by investigating the interactions of miRNAs and DNA methylation	Benincasa et al. (2019)
NCT01042301	120	T1DM	Interventional/Non-randomized	Completed	miRNA profiles were identified as biomarkers for T1DM at both the humoral and cellular levels	Nantes University Hospital (2015)
NCT02768935	20	MI	Interventional/Non-randomized	Unknown	Finding the miRNAs that help monocytes differentiate	Centre Hospitalier Universitaire de Nice (2022)
NCT02011100	28	T2DM, CVD	Interventional/Randomization	Completed	Effects of carnosine on CVD and diabetes risk factors; miRNAs that are linked to carnosine’s actions may lead to more effective treatment	Ukropec (2018)
NCT03890822	100	CAD	Observational/Prospective	Recruiting	inflammatory mediators and miRNAs play in diabetic people in progression of CAD	Stefanini (2019)
NCT04889053	1,400	T2DM, Vascular calcification (VC), Coronary artery calcification (CAC)	Observational/Prospective	Recruiting	Changes in miR-32 levels can help find VC early (prognostic biomarker)	University of South China (2021)
NCT05139914	50	Endothelial dysfunction	Interventional/Randomized	Not yet recruiting	Investigating how miRNA and other factors affect people with T2DM and the health of their blood vessels	Boston University (2023)

## Conclusion

DCM is a complex cardiovascular illness characterised by metabolic inefficiency, oxidative stress, inflammation, and fibrosis, and miRNAs play a crucial regulatory role in these pathological processes. The dysregulation of certain miRNAs contributes to cardiac apoptosis, hypertrophy, and decreased cardiac function, making them potential diagnostic indicators and therapeutic targets. Exosomal miRNAs, in particular, have demonstrated tremendous potential in enhancing heart function and metabolic regulation, opening up new pathways for early detection and intervention. Furthermore, ongoing clinical trials demonstrate the translational potential of miRNA-based treatments for CVD associated with diabetes. miRNAs play a major role in DCM by altering several biological factors and processes in various ways, as discussed above. Additionally, circulating miRNAs in the physiological system are highly stable, making them viable candidates as biomarkers for diagnosing, predicting, and combating cardiac illnesses. Several miRNAs, including miR-15a, miR-21, miR-24, miR-29b, miR-126, miR-150, miR-191, miR-29a, miR-30d, miR-124a, miR-146a, miR-375, miR-144, and miR-192, have been identified in the plasma or whole blood of individuals with diabetes, with miR-126 and miR-144 being strongly linked to the disease (Montgomery et al., 2011). Furthermore, plasma miR-503 has been linked to endothelial dysfunction and ischaemia in diabetes. Future research may provide additional insights into the differential expression of these miRNAs, perhaps increasing their utility as DCM biomarkers. Looking ahead, the future of miRNA-based diagnostics and therapies in DCM is dependent on the evolution of precision medicine through personalised treatment approaches. Researchers are working to develop anti-miRNAs and miRNA mimics as treatments for cardiomyopathy.

Antisense oligonucleotides, such as antagomiRs, have shown promise in suppressing overexpressed disease-causing microRNAs. In animal models, antagomiR-mediated silencing has been shown to be helpful in treating heart failure. For example, intravenous treatment with an antagomiR targeting miR-132 successfully inhibited native miR-132 expression in the heart, thereby preserving cardiac muscle from fibrosis, ventricular dilatation, hypertrophy, and heart failure, while increasing cardiac function.

While miRNAs hold tremendous potential as theranostic agents, their clinical application largely depends on the development of safe, efficient, and targeted delivery methods. Several advanced delivery systems, including Lipid Nanoparticles (LNPs), Polymeric Carriers, Viral Vectors, Modified Exosomes, Aptamer-Conjugated Systems, and Ultrasound-Targeted Microbubble Destruction (UTMD), have been developed to overcome challenges such as degradation, off-target effects, and limited bioavailability. LNPs have emerged as one of the most promising delivery platforms for nucleic acid-based therapeutics. They encapsulate miRNAs or their synthetic analogues (miRNA mimics or antagomirs) to protect them from nuclease-mediated degradation and enhance cellular uptake. Ionizable lipids, PEGylation, and optimised particle size improve circulation time and tissue-specific delivery, as demonstrated in mRNA vaccines (Hou et al., 2021). Another platform for miRNA delivery is the use of biodegradable polymers, such as poly (lactic-co-glycolic acid) (PLGA), polyethyleneimine (PEI), and chitosan-based nanoparticles. These carriers offer biocompatibility and tunable release profiles while facilitating endosomal escape and cytoplasmic release of the cargo. Recent studies have demonstrated the successful delivery of miR-21 using PLGA-based systems to restore cardiac function (Zhang et al., 2025). Moreover, Adeno-associated viruses (AAVs) and lentiviral vectors have demonstrated high transduction efficiency in cardiac tissues. AAV9, in particular, exhibits strong cardiomyotropic properties and has been used in preclinical studies to deliver miRNA regulators, such as anti-miR-25, in heart failure models, showing restored SERCA2a expression and improved cardiac function. However, safety concerns regarding immunogenicity and insertional mutagenesis remain to be addressed (Bilal et al., 2022). Recent advances allow surface modification of exosomes (e.g., by ligand decoration or electroporation) to enhance their targeting specificity toward cardiomyocytes or endothelial cells involved in DCM. Engineered exosomes carrying therapeutic microRNAs, such as miR-122, miR-146, and miR-335-5p, have shown promising outcomes in preclinical studies (Gorshkov et al., 2022).

However, challenges remain in the clinical translation of miRNA-based therapies. miRNAs are challenging to deliver through biological components because they are unstable in the bloodstream and lack selectivity. Each miRNA can regulate multiple genes and mRNA targets, making tailored delivery to the cardiovascular system particularly challenging. miRNA-based therapeutics targeting miR-122 and miR-34 are currently being tested for the treatment of hepatitis C and cancer, respectively, while antagomiRs such as miR-208/499 and miR-15/195 have reached the preclinical research stage for the management of heart failure and post-infarction remodelling (Szekely and Arbel, 2018). Collectively, these emerging delivery systems and technologies hold significant promise for advancing miRNA-based theranostic approaches in DCM by potentially improving efficacy, safety, and patient outcomes.

Despite these advances, more thorough research and clinical trials are needed to determine the efficacy and safety of miRNA-based therapeutics in cardiovascular medicine, particularly for DCM. Thus, identifying and characterising miRNAs and the pathways they regulate could serve as a foundation for developing new diagnostic and therapeutic tools for DCM management. The clinical implementation of miRNA-based techniques will require the integration of existing pharmacological treatments as well as the creation of stable, tissue-specific delivery mechanisms. As research advances, miRNA-based therapies have the potential to transform cardiovascular medicine by providing novel approaches to DCM prevention, diagnosis, and therapy.

## Glossary

AAV: Adeno-associated virus
ADAM: A Disintegrin and Metalloprotease
AGE: Advanced Glycation End product
AT II: Angiotensin II
AGO2: Argonaute RISC Catalytic Component 2
AMPK: AMP-activated protein kinase
AP: Activator protein
Bcl-2: B-cell lymphoma-2
CAD: Coronary Artery Disease
cAMP: Cyclic adenosine monophosphate
CD36: Cluster of Differentiation 36
CH: Cardiac hypertrophy
CHF: Congestive Heart Failure
CVDs: Cardiovascular disorders
DCM: Diabetic cardiomyopathy
DGCR8: DiGeorge Syndrome Critical Region 8
DM: Diabetes mellitus
DNMT: DNA methyltransferase
Ds-RNA: Double-stranded RNA
ECM: Extracellular matrix
FA: Fatty acids
FFA: Free fatty acids
FOXO: Forkhead box O
GLUT4: Glucose transporter type 4
HF: Heart failure
HFD: High fat diet
HFS-1: Heat-shock transcription factor-1
HG: High glucose
HO-1: Heme oxygenase-1
IGF-1: Insulin-like growth factor-1
IGFBPs: IGF binding proteins
IRS: Insulin receptor substrate
JNK: c-Jun NH2-terminal kinase
Keap1: Kelch-like ECH-associated protein 1
KO: Knockout
LDL: Low-density lipoprotein
MAPK: Mitogen-activated protein kinase
MDA: Malondialdehyde
mTOR: Mammalian target of rapamycin
MI: Myocardial Infarction
NCX: Na+-Ca^2+^exchanger
NF-κB: Nuclear factor kappa B
NQO1: NADPH quinone oxidoreductase
PI3K: Phosphatidylinositol 3-kinase
PKC: Protein kinase C
PPAR: Peroxisome proliferator activated receptor
RAAS: Renin Angiotensin Aldosterone System
RAGE: Receptor advanced glycated end product
RISC: RNA-induced silencing complex
RNS: Reactive nitrogen species
ROS: reactive oxygen species
RyR: ryanodine receptor
SERCA: sarcolemmal Ca^2+^ ATPase Channel
SGLT: Sodium-glucose co-transporter
shRNA: short hairpin RNA
ss-RNA: Single-stranded RNA
STZ: Streptozotocin
T1DM: Type 1 diabetes mellitus
TGF: Transforming growth factor
TIIA: Tanshinone IIA
TLR: toll-like receptors
TNF-α: Tumor necrosis factor alpha
VCAM: Vascular cell adhesion molecule
VH: Ventricular hypertrophy
VLDL: Very-low-density lipoprotein.
